# A Case of Mycosis Fungoides

**Published:** 1902-04

**Authors:** Alfred E. Diehl

**Affiliations:** Buffalo, N. Y.


### A Case of Mycosis Fungoides.

By ALFRED E. DIEHL, A. M., M. D., Buffalo, N. Y.

MYCOSIS fungoides, or more properly granuloma fungoides, was first described by Alibert in 1814. It is as a rule divided into three stages, as described by Bazin, but one or more of these stages may be absent in any case. The disease usually begins on the head, spreading downward, until the whole surface of the body may become involved, as sometimes happens in the early stage; later the disease becomes more circumscribed and is limited usually to the tumor formations.

The first stage is called the eczematous or premycosic stage. This is characterised by the formation of round or irregular patches of erythema, generally accompanied with severe itching. These patches may clear up in the center, forming circles or gyrate figures. Scratching, induced by the severe itching of the parts, leads to the production of vesicles, the exudation from which dries into crusts, giving the appearance of a true eczema. The patches of erythema may disappear from one locality, leaving behind a pigmented area, only to appear in some other situation. The duration of this stage varies from a few months to several years.

The second stage called by Bazin the "periode lichenoide," and by Köbner the "stadium infiltrationis," follows gradually and is characterised by the involvement of the entire thickness of the skin, which becomes infiltrated and stiff, and by the appearance of hard nodules, either in the patches of infiltration, or in otherwise healthy skin. These nodules may be bright red in color, or may differ in color but slightly from the surrounding skin, they may remain discrete, or they may become confluent. Like the patches of erythema, in the first stage, these nodules may come and go, leaving behind an apparently healthy skin, or a pigmented area. The itching is also severe, as in the first stage. The duration of the second stage varies also from a few months to several years.

The third stage is the stage of tumor formation, or the fungoid stage. The tumors formed in this stage vary in size from a pea to an orange; the smaller ones, as a rule, are bright red in color, or they may differ but little from the surrounding skin. The large tumors are lobulated and of a dark purplish color, the skin appearing tense and shining. The tumors have a peculiarity in that they will often disappear spontaneously in the course of a few days, leaving sometimes no mark of their presence or merely a slight pigmentation. As a result of some irritation or spontaneously, these tumors will finally ulcerate, giving forth a copious serous or bloody discharge and now present themselves as fungating tumors, having the appearance somewhat of a cut tomato, as Alibert described it. When these tumors ulcerate they are extremely liable to septic infections. The general health of the patient, which has remained comparatively good during the early stages, now becomes markedly impaired. There is anorexia, sleeplessness, fever, as a result of local infection and diarrhea, the patient dying eventually of exhaustion.

As previously stated, there may be an absence of one or more of the stages of the disease, and tumor formation may be the only stage observed, being then known as mycosis d'emblee. On the other hand, as sometimes occurs, all three stages may be present coincidently, as will be seen in the history of the appended case:

Mrs. E. M., widow, aged 52 years, had always been well; had no affection of the skin until eight years ago, when an eruption began on the right side of the neck, which was diagnosticated as an eczema, and treated as such ineffectually, the eruption gradually spreading over the entire body. Four years later some tumors made their appearance, being first noticed under the right arm, later other tumors appeared in the groin, and on the posterior surfaces of the legs. These tumors varied in size from a pea to a hen's egg, some of which disappeared leaving no trace; tumors had also appeared in the breasts and on the arms; these also had disappeared. The patient presented the following appearance when she came under my observation. Nearly the entire surface of her body was covered with a seborrheal condition, the arms particularly and the face and neck were affected with an eczematous eruption, presented many excoriations and crusts, and there was considerable exfoliation of the epidermis. Scattered over the surface of the body and particularly on the legs were several sharply circumscribed patches of a dark red color, elevated somewhat above the surrounding skin, varying in size from a quarter of an inch to an inch in diameter. These patches involved the entire thickness of the skin, and also showed scratch marks, presenting the second stage of the disease. The tumor formation presented the most striking appearance. On the left side of the neck was a tumor about the size of a hen's egg; the patient said that about three weeks before this tumor appeared, there had been a tumor on the opposite side of the neck, which began to decrease in size as the later tumor grew. On the right arm, above the elbow, was another tumor, about the size of an egg, firm in consistence, somewhat movable, which began about five weeks before. Patient said that there had been a tumor in the same situation previously, which had disappeared entirely. In each axilla, there were present three or four smaller tumors; scattered over the body were about a dozen small freely movable growths, varying in size from a pea to a pigeon's egg. On the right thigh, about six inches below Poupart's ligament, was a large lobulated tumor about the size of an orange, covered by a tense, purplish, shining skin. This tumor was very painful. The patient herself opened it with a penknife, thinking to ease the pain. About a pint of serous fluid exuded at the time of incision. The place of incision was the seat of a slough about two inches long by one-half inch wide. On the left leg was a large tumor, corresponding in size and situation to that on the right leg. Evidences of the disease were also seen on mucous membrane, both tonsils were enlarged, ulcerated and covered with a grayish slough; the soft palate was also ulcerated.

The patient was given arsenic in the form of Fowler's solution, hypodermatically, besides local applications to allay the itching. I was very forcibly impressed by the action of the arsenic, by the fact that each injection caused intense pain in the tumors, but nowhere else, the pain usually lasting three or four hours, necessitating the use of morphia. In the course of a week I was compelled to leave off the hypodermic medication on account of the pain. About a month after the patient came under my observation, the tumor on the left leg suddenly broke down and poured out a large quantity of bloody serum, and the tumor took the form of a dark red fungating mass, which discharged continually. The patient died two weeks later of exhaustion. An autopsy was not allowed. The only examination made was one of the blood, which showed a large increase in the number of eosinophiles; otherwise nothing of importance.

362 PEARL STREET.

